# Detecting miRNA Mentions and Relations in Biomedical Literature

**DOI:** 10.12688/f1000research.4591.3

**Published:** 2015-10-01

**Authors:** Shweta Bagewadi, Tamara Bobić, Martin Hofmann-Apitius, Juliane Fluck, Roman Klinger

**Affiliations:** 1 Fraunhofer SCAI, Bioinformatics, Schloss Birlinghoven, 53754, Sankt Augustin, Germany; 2 University of Bonn, B-IT, Dahlmannstr. 2, 53113 Bonn, Germany; 3 Hasso Plattner Institute Potsdam, Prof.-Dr.-Helmert-Str. 2-3, 14482 Potsdam, Potsdam, Germany; 4 Semantic Computing Group, CIT-EC, Bielefeld University, 33615 Bielefeld, Germany

**Keywords:** MicroRNAs, prediction algorithms, corpus

## Abstract

**Introduction:** MicroRNAs (miRNAs) have demonstrated their potential as post-transcriptional gene expression regulators, participating in a wide spectrum of regulatory events such as apoptosis, differentiation, and stress response. Apart from the role of miRNAs in normal physiology, their dysregulation is implicated in a vast array of diseases. Dissection of miRNA-related associations are valuable for contemplating their mechanism in diseases, leading to the discovery of novel miRNAs for disease prognosis, diagnosis, and therapy.

**Motivation:** Apart from databases and prediction tools, miRNA-related information is largely available as unstructured text. Manual retrieval of these associations can be labor-intensive due to steadily growing number of publications. Additionally, most of the published miRNA entity recognition methods are keyword based, further subjected to manual inspection for retrieval of relations. Despite the fact that several databases host miRNA-associations derived from text, lower sensitivity and lack of published details for miRNA entity recognition and associated relations identification has motivated the need for developing comprehensive methods that are freely available for the scientific community. Additionally, the lack of a standard corpus for miRNA-relations has caused difficulty in evaluating the available systems.

We propose methods to automatically extract mentions of miRNAs, species, genes/proteins, disease, and relations from scientific literature. Our generated corpora, along with dictionaries, and miRNA regular expression are freely available for academic purposes. To our knowledge, these resources are the most comprehensive developed so far.

**Results:** The identification of specific miRNA mentions reaches a recall of 0.94 and precision of 0.93.  Extraction of miRNA-disease and miRNA-gene relations lead to an *F _1_* score of up to 0.76. A comparison of the information extracted by our approach to the databases *miR2Disease* and *miRSel* for the extraction of Alzheimer's disease related relations shows the capability of our proposed methods in identifying correct relations with improved sensitivity. The published resources and described methods can help the researchers for maximal retrieval of miRNA-relations and generation of miRNA-regulatory networks.

**Availability:** The training and test corpora, annotation guidelines, developed dictionaries, and supplementary files are available at http://www.scai.fraunhofer.de/mirna-corpora.html

## Introduction

Functionally important non-coding RNAs (ncRNAs) are now better understood with the progress of high-throughput technologies. Discovery of the major class of ncRNAs, microRNAs (miRNAs ^1^) has further facilitated the molecular aspects of biomedical research.

MicroRNAs are a large group of small endogenous single-stranded non-coding RNAs (17–22nt long) found in eukaryotic cells. They post-transcriptionally regulate gene expression of specific mRNAs by degradation, translational inhibition, or destabilization of the targets (transcripts of protein-coding genes) ^2^. Esquela-Kerscher *et al.* have reported on miRNAs involvement in almost every regulation aspect of biological processes such as apoptosis, and stress response ^3^. Wubin *et al.* demonstrated that miR-29a regulatory circuitry plays an important role in epididymal development and its functions ^4^. Additionally, tissue-specificity of miRNAs has been shown to provide a better clue of their fundamental roles in normal physiology ^5^.

Dysregulation of miRNAs and their ability to regulate repertoires of genes (as well as co-ordinate multiple biological pathways) has been linked to several diseases ^6, 7^. One example is chronic lymphocytic leukemia where (in about 68% of the cases) miRNA genes ( *miR15* and *miR16*) are missing or down-regulated ^8^. Thus, uncovering the relations between miRNAs and diseases as well as genes/proteins is crucial for our understanding of miRNA regulatory mechanisms for diagnosis and therapy ^9, 10^.

Several databases, prediction algorithms and tools are available, providing insight into miRNA-disease and miRNA-mRNA associations. Although the detailed target recognition mechanism is still elusive, several algorithms attempt to predict miRNA targets. However, a limited precision of 0.50 and recall of 0.12 has been reported when evaluated against proteomics supported miRNA targets ^11^. Despite the fact that these resources provide insight into miRNA-associated relationships, the majority of relations are scattered as unstructured text in scientific publications ^12^. Figure 1 shows the growth of publications in MEDLINE and in addition depicts the normalized growth of publications that reference the keyword “microRNA”.

**Figure 1.**
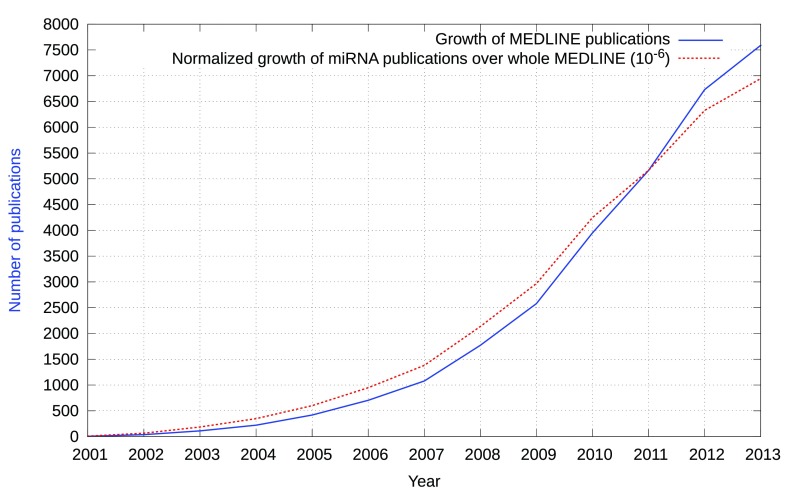
Growth of miRNA-related publications in comparison with the growth of MEDLINE. The dotted line points out the relative increase of miRNA-related publications per year in comparison to the growth of MEDLINE (as of 31 December, 2013).

Some databases such as *miR2Disease* and *PhenomiR* store manually extracted relations from literature. The *miR2Disease* database ^13^ contains information about miRNA-disease relationships with 3273 entries (as of the last update on March 14, 2011). *PhenomiR* ^14^ is a database on miRNA-related phenotypes extracted from published experiments. It consists of 675 unique miRNAs, 145 diseases, and 98 bioprocesses from 365 articles (Version 2.0, last updated on February 2011). *TarBase* ^11^ hosts more than 6500 experimentally validated miRNA targets extracted from literature.

However, manual retrieval of relevant articles and extraction of relation mentions from them is labor-intensive. A solution is to use text-mining techniques. Moreover, the vast majority of the research in this direction is mainly focused around extraction of protein-protein interactions ^15^. On the contrary, miRNA relation extraction is still naive. The shift of focus towards identification of miRNA-relations is slowly establishing with the rise in systems approaches to investigate complex diseases. The manually curated database *miRTarbase* ^16^ incorporates such text-mining techniques to retrieve miRNA-related articles. Recently, the *miRCancer* database has been constructed using a rule-based approach to extract miRNA-cancer associations from text ^17^. As of June 14, 2014, this database contains 2271 associations between 38562 miRNAs and 161 human cancers from 1478 articles.

### Related work

Text-mining technologies are established for a variety of applications. For instance, the BioCreative competition ^18, 19^ and BioNLP Shared Task ^20– 22^ series have been conducted to benchmark text mining techniques for gene mention identification, protein-protein relation extraction and event extraction, among others.

To our knowledge, only limited work has been carried out in the area of miRNA-related text-mining. Murray *et al.* considered miRNA-gene associations from PubMed database using semantic search techniques ^23^. For their analysis, experimentally derived datasets were examined, combined with network analysis and ontological enrichment. Regular expressions were used to detect miRNA mentions. The authors claim to have optimized the approach to reach 100% accuracy and recall for detecting miRNAs mentions as in *miRBase*. Relations were identified based on a manually curated rule set. The authors extracted 1165 associations between 270 miRNAs and 581 genes from the whole MEDLINE.

The freely available *miRSel* ^12^ database integrates automatically extracted miRNA-target relationships from PubMed abstracts. A set of regular expressions is used for miRNA recognition that matches all *miRBase* synonyms and generic occurrences. The authors reach a recall of 0.96 and precision of 1.0 on 50 manually annotated abstracts for miRNA mention identification. Further, the relations between miRNA and genes were extracted at sentence level employing a rule-based approach. They evaluated on 89 sentences from 50 abstracts resulting in a recall of 0.90 and precision of 0.65. Currently, it hosts 3690 miRNA-gene interactions ^11^.

Since the miRNA naming convention has been formalized very early in comparison to other biological entities such as genes and proteins, applying text-mining approaches is relatively simple ^17^. Thus, most of the previously applied text mining approaches for miRNA detection has been based on keywords. *miRCancer* uses keywords to obtain abstracts from PubMed, further miRNA entities have been identified using regular expressions based on prefix and suffix variations. Similarly, *miRWalk* database uses keyword search approach to download abstracts and applies a curated dictionary (compiled from six databases) for miRNA identification of human, rat, and mouse species ^24^. *TarBase*, *miR2Disease*, *miRTarBase*, and several others have followed related search strategies. However, several authors still tend to use naming variations for acronyms, abbreviations, nested representations, *etc.* for listing miRNAs. Additionally, in contrast to the previous text-mining approaches focusing purely on miRNA gene relations, we extend the information extraction approach additionally to retrieve miRNA-disease relations. Furthermore, we evaluate our approach using a larger corpus to achieve robustness. We differentiate between actual miRNA mentions (refered to as S pecific miRNAs) and co-referencing miRNAs (N on-S pecific miRNAs), which could in addition enhance keyword search. We evaluated three different relation extraction approaches, namely co-occurrence, tri-occurrence and machine learning based methods.

To support further research, our corpora are made publicly available in an established XML format as proposed by Pyysalo *et al.* ^25^, as well as the regular expressions used for miRNAs named entity recognition. In addition, our dictionary for trigger term detection and general miRNA mention identification are made available. To our knowledge, the annotated corpora as well as the information extraction resources are the most comprehensive developed so far.

## Methods

### Data curation and corpus selection

***Named entities annotation.*** Mentions of miRNAs consisting of keywords (case-insensitive and not containing any suffixed numerical identifier) such as *“Micro-RNAs”* or *“miRs”* are annotated as N on-S pecific miRNA. Names of particular miRNAs such as *miRNA-101*, suffixed with numerical identifiers are labeled as S pecific miRNA. Numerical identifiers (separated by delimiters such as “,”, “/”, and “and”) occurring as part of specific miRNA mentions are annotated as a single entity. Box 1 depicts the annotation of specific miRNA mentions (including an example for part mentions). In addition, D isease, G ene/P rotein, S pecies, and R elation T rigger are annotated. The detailed annotation guideline for annotating specific miRNA mentions is available as a supplementary file.

Box 1. **Example of miRNAs annotations.**

Here “ *-181b*”, and “ *-181c*” are the part mentions annotated as a single entity along with “ *miR-181a*” in box. A non-specific miRNA mention is shown in italics.

Interesting results were obtained from miR-181a, -181b, and -181c. These set of brain-enriched *miRNAs* are down-regulated in glioblastoma. However, miR-222, and miR-128 are strongly up-regulated.

Mentions of disease names, disease abbreviations, signs, deficiencies, physiological dysfunction, disease symptoms, disorders, abnormalities, or organ damages are annotated as D isease. Only disease nouns were considered, adjective terms such as “ *Diabetic patients*” are not marked; however, specific adjectives that can be treated as nouns were marked, e.g. “Parkinson’s disease patients”. Mentions referring to proteins/genes which are either single word ( *e.g. “trypsin”*), multi-word, gene symbols ( *e.g. “SMN”*), or complex names (including of hyphens, slashes, Greek letters, Roman or Arabic numerals) are annotated as G ene/P rotein. Only those organisms that are having published miRNA sequences and annotations represented in *miRBase* database are labeled as S pecies. Any verb, noun, verb phrase, or noun phrase associating miRNA mention to either labeled disease or gene/protein term is annotated as R elation T rigger.

***Relations annotation.*** We restrict the relationship extraction to sentence level and four different interacting entity pairs: S pecific miRNA-D isease (SpMiR-D), S pecific miRNA-G ene/P rotein (SpMiR-GP), N on-S pecific miRNA-D isease (NonSpMiR-D), and N on-S pecific miRNA-G ene/P rotein (NonSpMiR-GP). Relevant triples, an interacting pair (from one of the above-mentioned) co-occurring with a R elation T rigger in a sentence are defined to form a relation and can belong to one of the four above-mentioned Relation classes. On the contrary, if an interacting pair does not co-occur with any R elation T rigger then we do not tag such pair as a relation.

The annotation has been performed using Knowtator ^26^ integrated within the Protégé framework ^27^.

***Corpus selection, annotation and properties.*** We develop a new corpus based on MEDLINE, annotated with miRNA mentions and relations. Shah *et al.* ^28^ showed that abstracts provide a comprehensive description of key results obtained from a study, whereas full text is a better source for biological relevant data. Thus, we choose to build the corpus for abstracts only. Out of 27001 abstracts retrieved using the keyword “miRNA”, 201 were randomly selected as training and 100 as test corpus. Two annotators performed the annotation. The first annotator annotated the training corpus iteratively to develop guidelines and built the consensus annotation. The second annotator followed these guidelines and annotated the same corpus. Disagreeing instances were harmonized by both the annotators through manual inspection for correctness and its adherence to the guidelines. Any changes to the guidelines were made if needed. During the harmonization process only the non-overlapping instances between the two annotators were investigated. Decisions were based on the rule that only noun forms were to be marked (specific adjectives that can be treated as nouns were also considered). In case of partial matches, where conflicting parts could be interpreted as an adjective were not resolved. For example, in “chronic inflammation”, marking either “chronic inflammation” or just “inflammation” were considered correct. Table 1 provides the inter-annotator agreement (measured as *F*_1_, for both exact and boundary match, and Cohen’s *κ*) for the test corpus. Exact string match occurs only when both the annotators annotate identical strings, whereas in partial match fraction of the string has been annotated by either of the annotators. It is evident ( *cf.* Table 1) that in almost all cases partial match performs better than exact string match, indicating variations in span of mentioned entities. An example annotation is shown in Box 1.

**Table 1.** Inter-annotator agreement scores for the test corpus.

Annotation Class	*F*_1_ (Exact Match)	*F*_1_ (Partial Match)	*κ*
Non-specific MiRNAs	0.9985	0.9985	0.996
Specific MiRNAs	0.9545	0.9779	0.916
Genes/Proteins	0.8343	0.8705	0.752
Diseases	0.8270	0.9575	0.853
Species	0.9329	0.9437	0.875
Relation Triggers	0.8441	0.9543	0.798

Table 2 shows the number of annotated concepts in the training and test corpora for each entity class and the count for manually extracted relations (triplets), categorized for different interacting entity pairs. Table 3 provides the overall statistics of the published corpora (additional information about the corpus is given in the README supplementary file).

**Table 2.** Manually annotated entities statistics. Counts of manually annotated entities in the training and the test corpora as well as annotated sentences describing relations.

Annotation Class	Corpus	
	Training	Test
Non-specific MiRNAs	1170	336
Specific MiRNAs	529	376
Genes/Proteins	734	324
Diseases	1522	640
Species	546	182
Relation Triggers	1335	625
SpMiR-D	171	127
SpMiR-GP	195	123
NonSpMiR-D	124	54
NonSpMiR-GP	77	16

**Table 3.** Statistics of the published miRNA corpora.

Occurrences in the corpus	Training	Test
Sentences	1864	780
Entities	5836	2483
Entity pairs	2001	868
Positive entity pairs	567	320
Negative entity pairs	1434	548

### Automated named entity recognition

For identification of specific miRNA mentions in text ( *cf.* Table 4), we developed regular expression patterns using manual annotations of miRNA mentions as the basis. Similarly, a dictionary has been generated for general miRNA recognition. The regular expression patterns are represented in the format as defined by Oualline *et al.* ^29^. For simplicity and reusability, several aliases are defined ( *cf.* Table 5) to be used in the final regular expression patterns for specific miRNA identification, given in Table 4. Detected entities are resolved to a unique miRNA name and disambiguated to adhere to standard naming conventions as authors use several morphological variants to report the same miRNA term. For example, miR-107 can be represented as miRNA-107, Micro RNA-107, MicroRNA 107, has-mir-107, mir-107/108, micro RNA 107 and 108, micro RNA (miR) 107 and so on. Thus, the identified miRNA entity has been resolved to its base form ( *e. g. hsa-microRNA-21* to *hsa-mir-21* and *microRNA 101* to *mir-101*) following the miRBase naming convention. Manual inspection of the test corpus for species distribution revealed that 71% of the documents belonged to human, followed by mouse (15%), rat (8%). Pig has 2 abstracts, zebrafish, HIV-1, HSV-1, and *Caenorhabditis elegans* 1 each ( *cf.* Supplementary Figure A for the distribution). Thus, we assumed that most of the abstracts belonged to human and resolved the identified miRNA entities to human identifier in miRBase. Unique miRNA terms are mapped to human *miRBase* database identifiers through the mirMaid Restful web service. For those names where we do not retrieve any database identifiers, we fall back to another organism mention found in the abstract (if any), using the NCBI taxonomy dictionary (see below) ( *cf.* Supplementary Figure B), otherwise we retain the unique normalized name ( *cf.* Box 2).

**Table 4.** Regular expression patterns used for miRNAs identification. Aliases used to form the final regular expression, see Table 5, are highlighted in bold.

Regular expression patterns	Description	Example of identified text
**(Pref+(Lin,Let))**	Detection of *Lin* and *Let* variations of miRNAs	lin-4; hsa-let-7a-1
(Pref+(miRNA, Onco)(S*Tail)(Sep Tail)*)	MiRNAs mentions for different separators	hsa-mir-21/22; Oncomir-17∼92
(Pref+(miRNA, Onco) S*(D(Z([/]Z)*)+) ([\,] S*? (Pref+(miRNA, Onco) S*(D(Z([/]Z)*)+)*)))	Multiple miRNA mentions occurring progressively	miR-17b, -1a; hsa-miR-21,22, and hsa-miR-17

**Table 5.** MiRNAs regex aliases. Aliases used in regular expression patterns for miRNAs identification (highlighted in bold).

Description	Alias	Regular Expression Pattern
Digit sequences	**D**	(\d?\d*)
Admissible hypens with a trailing space	**Z**	([\-]?[\-]*)
Admissible hypens with a leading space	**S**	([\-]?[\-]*)
3-letter prefix for human followed by a hyphen	**Pref**	([hH][sS][aA][\-])
Non-specific miRNA mentions	**miRNA**	([mM][iI]([cC][rR][oO])+[rR]([nN][aA]s+)+)
*Let-7* miRNA mention	**Let**	([lL][eE][tT] S*[7]?\l+)
*Lin-4* miRNA mention	**Lin**	([lL][iI][nN] S*[4]?\l+)
*Oncomir* miRNA mention	**Onco**	([oO][nN][cC][oO][mM][iI][rR])
Admissible tilde and word boundaries	**Cluster**	(∼[\b]-[\b]-*)
Admissible hyphen and separator *and* and *comma*	**Sep**	( S*((and?, **S**,\/,)? S*)+)
Admissible combination of upper and lower case alphabets	**UL**	(?\l?\l+,?\u?\u+)
Admissible alpha-numerical identifiers in specific miRNA mentions	**AN**	( **UL**((/, *and*, **D**+)? **UL**)+)
Admissible alpha-numerical identifiers in oncomir mentions	**Tail**	( **D**( **AN** **Cluster**+,\- **D** **AN**+)+)

Box 2. **Un-normalized and normalized entities that are mapped to miRBase identifiers. Here MIR0000007, MIR0000008, and MIR0000005 are internal identifiers used by ProMiner.**

MIR0000007:MIMAT0015092@MIRBASE|MI0000002@MIRBASE|cel-lin-4|lin-4MIR0000008: miR-171|microRNA 171MIR0000005:MIMAT0000416@MIRBASE|has-miR-1|miRNA-1

We detect S pecies with a dictionary-based approach. The built dictionary consists of all the concepts from the NCBI taxonomy corresponding to only those organisms mentioned in *miRBase*.

Similarly, for identification of D isease and G ene/P rotein mentions in text we adapted a dictionary-based approach. To detect D isease, we apply three dictionaries: MeSH, MedDRA ^30^ and *Allie*. For G ene/P rotein, a dictionary ^31^ based on SwissProt, EntrezGene, and HGNC is included. Gene synonyms which could be potentially tagged as miRNAs are removed to overcome redundancy. For example, genes encoding microRNA, *hsa-mir-21* are named as *miR-21*, *miRNA21* and *hsa-mir-21*, the gene symbol of *MIR16 membrane interacting protein of RGS16* is *MIR16*, which can represent a miRNA mention.

The relation trigger dictionary comprises of all interaction terms from the training corpus. After reviewing the training corpus for relation trigger terms, we retrieved not one but many variants of the same relation trigger occurring in alternative verb-phrase groups. For example, “change in expression” can be represented as one of the following verb-phrases: Change MicroRNA-21 Expression, Expression of caveolin-1 was changed, Change in high levels of high-mobility group A2 expression, change of the let-7e and miR-23a/b expression, expression of miR-199b-5p in the non-metastatic cases was significantly changed, etc. To allow flexibility for capturing relation trigger along with its variants spanning over different phrase length, we first manually represented all the relations in its root form, such as “regulate expression” to “regulate” ( *cf.* Relation_Dictionary.txt file in Dataset 1). The base form has been extended manually to different spelling variants, *e.g.* regulate to regulatory, regulation, etc., the detailed listing of variants is provided in Word_variations.txt in Dataset 1. Not all combinations of the root forms are logical; target and up-regulation terms cannot be combined to form a relation trigger. Thus, we additionally defined a set of relation combinations that are allowed (see Permutation_terms.txt in Dataset 1 for all combinations).

For all named entity recognition performed, the dictionary-based system ProMiner ^31^ is used. Supplementary Table A ( Dataset 1) provides a quantitative estimate of the entities available in the dictionaries used in this work.

### Relation extraction

We consider three approaches for addressing automatic extraction of interacting entity pairs from free text, described in the following.

The co-occurrence approach serves as a baseline. Assuming all interactions to be present in isolated sentences, this approach is complete but may be limited in precision. Reducing the number of false positives can be achieved by filtering with the dictionary of relation triggers occurring in the same sentence. The rationale behind this filter is that the interaction is more likely to be described if such a term is present (we refer to this as tri-occurrence).

To increase the precision, we use a machine learning-based approach formulating the relation detection as a binary classification problem: each instance (consisting of a pair of entities) is classified either as not-containing a relation or belonging to one of the four-relation classes. Our system uses lexical and dependency parsing features. We evaluate linear support vector machines (SVM) ^32^ as implemented in the LibSVM library, as well as LibLINEAR, a specialized implementation for processing large data sets ^33^, and naive Bayes classifiers ^34^. For more details, we refer to Bobić *et al.* ^35^.

Lexical features capture characteristics of tokens around the inspected pair of entities. The sentence text can roughly be divided into three parts: text between the entities, text before the entities, and text after the entities. Stemming ^36^ and entity blinding is performed to improve generalization. Features are bag-of-words and bi, tri, and quadri-gram based. This feature setting follows Yu *et al.* and Yang *et al.* ^37, 38^. The presence of relation triggers is also taken into account, using the previously described manually generated list. Next to lexical features, dependency parsing (created using Stanford parser) provides an insight into the entire grammatical structure of the sentence ^39^ and was performed using the Stanford CoreNLP library ( http://nlp.stanford.edu/software/corenlp.shtml). Deep parsing follows the shortest dependency path hypothesis ^40^. We analyzed the vertices *v* (tokens from the sentence) in the dependency tree from a lexical (text of the token) and syntactical (POS tag) perspective. Edges *e* in the tree correspond to the information about the grammatical relations between the vertices. Extracting relevant information from the dependency parse tree is usually done following the shortest dependency path hypothesis ^40^. Lexical and syntactical *e-walks* and *v-walks* on the shortest path are created by alternating sequence of vertices and edges, with the length of 3. We capture the information about the common ancestor vertex, in addition to checking whether the ancestor node represents a verb form ( *e.g.* POS tag could be VB, VBZ, VBD, etc.). Finally, the length of the shortest path (number of edges) between the entities is considered as a numerical feature.

## Results and discussion

Version 2. Manually annotated miRNA-disease and miRNA-gene interaction corpora Please see README.txt in the zip file for precise details about the corpus and supplementary files. The updated zip file contains new files (Permutation_terms.txt, Non-Specific_miRNAs_Dictionary.txt and Word_variations.txt) and Table A has been updated. Click here for additional data file. Copyright: © 2015 Bagewadi S et al. 2015 Data associated with the article are available under the terms of the Creative Commons Attribution-NonCommercial Licence, which permits non-commercial use, distribution, and reproduction in any medium, provided the original data is properly cited.

In the following, we present results for named entity recognition and relation extraction. This section concludes with two use-case analyses.

### Performance evaluation of named entity recognition

Among the 201 abstracts present in the training corpus, 82% contained general miRNA mentions, in comparison to specific miRNAs with 45%. In Table 6, results for miRNA entity recognition are reported. Non-specific miRNA recognition is close to perfect. Specific miRNA mention recognition has an *F*_1_ measure of 0.94.

**Table 6.** Evaluation results for miRNA entity classes. Here only complete match results are presented. The performance of named entity recognition is evaluated using recall ( *R*), precision ( *P*) and *F*_1_ score.

Entity Class	*R*	*P*	*F*_1_	*R*	*P*	*F*_1_
	Training Corpus			Test Corpus		
Non-specific MiRNAs	1.000	0.995	0.997	1.000	0.997	0.999
Specific MiRNAs	0.921	0.928	0.924	0.936	0.934	0.935
Relation Triggers	0.864	0.885	0.874	0.790	0.842	0.815

For disease mention recognition, combined dictionaries, based on three established resources, resulted in 0.79 and 0.69 *F*_1_ score for the training and test corpus respectively. The low score for disease identification could be due to the variation in disease mentions, such as multi-word, synonym combination, nested names, etc. However, the partial matches result for diseases reported 0.88 of *F*_1_, providing the possibility for detection of similar text strings for better recall ( *cf.* Supplementary Table B in Dataset 1). Genes/proteins dictionary showed a performance of 0.84 and 0.85 of *F*_1_ in training and test corpus respectively.

The evaluation of the relation trigger dictionary ( *cf.* Table 6) suggests that it covers a substantial part of the vocabulary with recall of 0.86 for the training and 0.79 for the test corpus.

### Relation extraction

 We queried MEDLINE for “miRNA and Epilepsy” documents, among which 16 documents containing miRNA-related relations were manually selected ( *cf.* Supplementary Figure C for the detailed distribution statistics). To avoid any biased approach we choose Epilepsy disease domain. Manual inspection of these articles revealed 11.5% of miRNA-related associations occur outside the sentence level. Thus, our work focused on relations at sentence level. Sentences in which co-occurring entity pairs do not participate in any relation are tagged as *false*. A comparison of the different relation extraction approaches is shown in Figure 2. Supplementary Table D in Dataset 1 provides statistical details of the applied approaches given in Figure 2. If all the entities are correctly identified then co-occurrence based approach leads to 100% recall for relation extraction. The recall is not diminished using the tri-occurrence approach, as the true entity pairs remain constant, while the precision increases between 4pp (percentage points) and 17pp when compared to the co-occurrence based approach, reducing false positives ( *cf.* Figure 2). However, overall the precision reaches less than 60%. In our work, we assume that all the entities have been identified giving a recall of 100% for both co-occurrence and tri-occurrence based approaches. Using the machine-learning based classification, precision is increased up to 76% for specific miRNA-gene relations for both LibLINEAR and LibSVM methods, although Naïve Bayes is not far behind. Similarly, these two methods performed nearly the same for specific miRNAs-disease relations, the *F*_1_ measure is not substantially different but a trade-off between precision and recall can be observed. An increase in *F*_1_ measure is observed for non-specific miRNA relations when Naïve Bayes method is applied, out performing other strategies. Nevertheless, preference of the method highly depends on the compromise one chooses, whether better recall or precision. Overall, better recall and acceptable precision can be achieved with tri-occurrence method.

**Figure 2.**
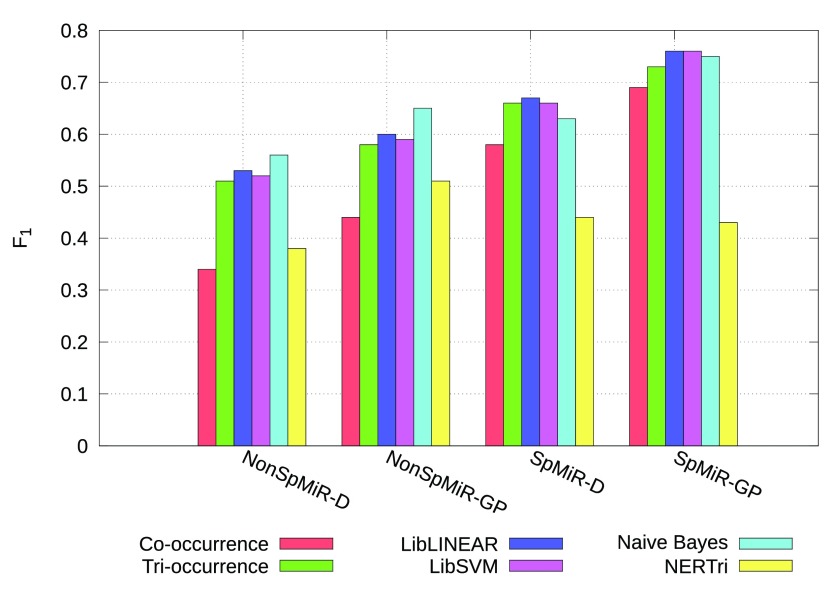
Comparison of different relation extraction approaches. On the x-axis, different entity pair relations are represented as SpMiR-D for S pecific miRNA-D isease, SpMiR-GP for S pecific miRNA-G ene/P rotein, NonSpMiR-D for N on-S pecific miRNA-D isease, and NonSpMiR-GP for N on-S pecific miRNA-G ene/P rotein.

Most relation extraction approaches are dependent on the performance of named entity recognition. The impact of error propagation coming from automated entity recognizers is evaluated by applying the tri-occurrence method on the automatically annotated training and test corpus, here termed as “NERTri”. Compared to the results on the gold standard entity annotation a drop of 13 pp for NonSpMiR-D, 7pp for NonSpMiR-GP, 22pp for SpMiR-D, and 30pp for SpMiR-GP in *F*_1_ is observed for the test corpus. Overall performance of the NERTri approach on training and test corpus is detailed in Supplementary Table C in Dataset 1.

## Use case analysis

For the impact analysis of the proposed approach, we compare the extracted information with two databases, namely *miR2Disease* and *miRSel*. We focus on relations and articles concerning Alzheimer’s disease.

Alzheimer’s disease (AD) is ranked sixth for causing deaths in major developed countries ^41^. It affects not only individuals but also incurs a high cost to the society. Recently, miRNAs have shown close associations with AD pathophysiology ^42, 43^. Increasing the need to identify new therapeutic targets for AD, after major set backs due to failed drugs, motivates the need to look in this direction. *In silico* methods, such as the one proposed in this work, can aid in building miRNA-regulatory networks specific to AD, for further analysis such as identifying the mechanisms, sub-networks, and key targets.

### Extracting miRNA-Alzheimer’s disease relations from full MEDLINE

The database *miR2Disease* is queried to return all miRNA-disease relations occurring in Alzheimer’s disease. For comparison, we retrieved miRNA-disease relations from MEDLINE using NERTri approach, resulting in 41 abstracts containing 159 relations. Obtained triplets have been manually curated to remove 51 false positives. False negatives have not been accounted, which may result in loss of information ( *cf.* Relation extraction section). Comparison between the relations obtained from *miR2Disease* and NERTri are summarized in Table 7. The *miR2Disease* database returns 28 evidential statements from 9 articles. Among these, only 14 evidences are present in abstracts. Moreover, 16 evidences are extracted from one full text document ^44^. Only two evidences are identified at abstract level among these 16 evidences. Overall, 26 miRNAs identified by *miR2Disease* refer to Alzheimer’s disease. Therefore, our text-based extraction proposes approximately three times more relations than the database provides.

**Table 7.** miR2Disease database comparison. MiRNA-Alzheimer’s disease relation retrieved from MEDLINE and in *miR2Disease* database.

	miR2Disease	NERTri	True Positives in NERTri	NERTri and miR2Disease Overlap
Publications	9	41	36	8
Relations	28	159	108	11
Evidences (abstracts)	14	159	108	10
Unique miRNAs	26	46	40	16

The analysis of 17 false negative relations which are in the database but not found by our approach shows that most of the relations could be found only in full text and that the automatic system misses four miRNA-Alzheimer’s disease relations from abstracts. Manual inspection reveals that in three out of these missing four evidences the disease name is not mentioned in the sentence (relation occurred at co-reference level).

### Extraction of miRNA-gene relations for Alzheimer’s diseases from full MEDLINE

Here we compare the performance of our relation detection NERTri with another text-mining database, *miRSel*. For comparison, 100 abstracts from PubMed were retrieved using the query *“alzheimer disease”[MeSH Terms] OR (“alzheimer disease”[All Fields] OR “alzheimer”[All Fields]) AND (“micrornas”[MeSH Terms] OR “micrornas”[All Fields] OR “microrna”[All Fields]) AND (“2001/01/01”[PDAT]:“2013/7/4”[PDAT])*. Manual inspection of these articles leads to 184 miRNA-gene relations, at sentence level, ( Table 8) in 37 abstracts.

**Table 8.** miRSel database comparison. Comparison of miRNA-gene relations retrieval for Alzheimer’s disease in MEDLINE.

Approach	Articles	Relations
PubMed Query (“Alzheimer AND miRNA”)	100	NA
PubMed Query with relations at sentence level	37	184
PubMed Query ∩ NERTri	28	140
PubMed Query ∩ miRSel	12	56
NERTri ∩ miRSel	14	22

NERTri approach was able to identify 140 of these found relations in 28 abstracts. Among the 37 abstracts from the PubMed query, *miRSel* contained only 12 abstracts with 56 miRNA-gene relations (cf. Table 8). False negatives in our approach when compared with *miRSel* could not be directly identified as the database is not downloadable and searchable for disease specific relations. However, low intersection between *miRSel* and NERTri can be observed.

In summary, our approach provides AD related gene-microRNA relations from PubMed which have not been available in the database before.

Overall, the results are promising when compared with the *miR2Disease* and *miRSel* databases and indicate that we can extend the databases to a large extent with new relations. Such an approach makes it much easier to keep databases up to date. Nevertheless full text processing would most certainly increase the recall of automatic processing.

## Conclusion and future work

In this work, we proposed approaches for identification of relations between miRNAs and other named entities such as diseases, and genes/proteins from biomedical literature. In addition, details of named entity recognition for all the above entity classes have been described. We distinguished two types of miRNA mentions, namely Specific (with numerical identifiers) and Non-Specific (without numerical identifiers). Non-specific miRNAs entity recognition has enabled us to achieve better recall and precision in document retrieval. Three different relation extraction approaches are compared, showing that the tri-occurrence based approach should be the first reliable choice among all others. The tri-occurrence based approach is comparable to a machine learning-based method but considerably faster. In comparison to two well-established databases, we have shown that additional useful information can be extracted from MEDLINE using our proposed methods.

To best of our knowledge, this is the first work where manually annotated corpora containing information about miRNAs and miRNA-relations are published. Moreover, the corpora and methods provided represent useful basis and tools for extracting the information about miRNAs-associations from literature. This work serves as an important benchmark for current and future approaches in automatic identification of miRNA relations. It provides the basis for building a knowledge-based approach to model regulatory networks for identification of deregulated miRNAs and genes/proteins.

The proposed methods encourage the extension of this work to full-text articles, to elucidate many more relations from Biomedical literature. Non-specific miRNA mention identification could prove highly beneficial for co-reference resolution in full-text articles, in addition to abstracts. Proposed machine-learning approaches could be applied to only tri-occurrence based instances for reducing the false positive rates. Extending the current approach to other model organisms such as mouse, and rat could be helpful in revealing important relations for translational research. Inclusion of additional named entities such as drugs, pathways, etc. could lead to an interesting approach for detection of putative therapeutic or diagnostic drug targets through a gene-regulatory network generated from identified relations.

## Data availability

The data referenced by this article are under copyright with the following copyright statement: Copyright: © 2015 Bagewadi S et al.

Data associated with the article are available under the terms of the Creative Commons Attribution-NonCommercial Licence, which permits non-commercial use, distribution, and reproduction in any medium, provided the original data is properly cited. 

Corpora availability: http://www.scai.fraunhofer.de/mirnacorpora.html

**Archived corpora at time of publication:** F1000Research: Dataset 1. Version 2. Manually annotated miRNA-disease and miRNA-gene interaction corpora, 10.5256/f1000research.4591.d40643 ^45^

## Figures and Tables

**Supplementary Figure A.**
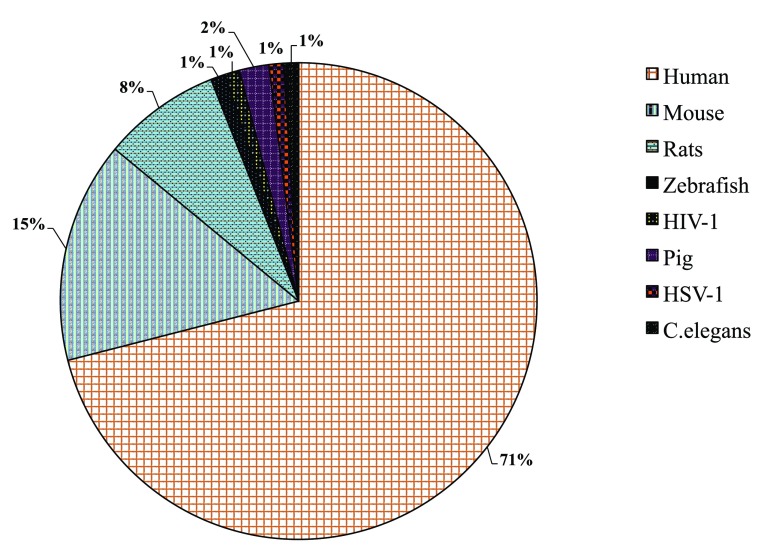
Distribution of organism mentions in training corpus.

**Supplementary Figure B.**
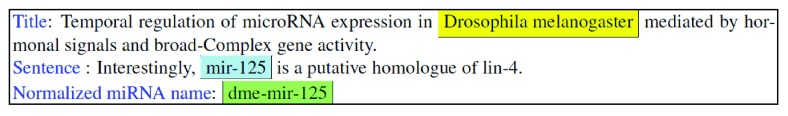
A screenshot example of how we handle other organism miRNA normalization. There is no miR-125 entry related to human in miRBASE. Since the abstract mentions *Drosophila melanogaster* in the title, the miRNA is normalized to dme-mir-125.

**Supplementary Figure C.**
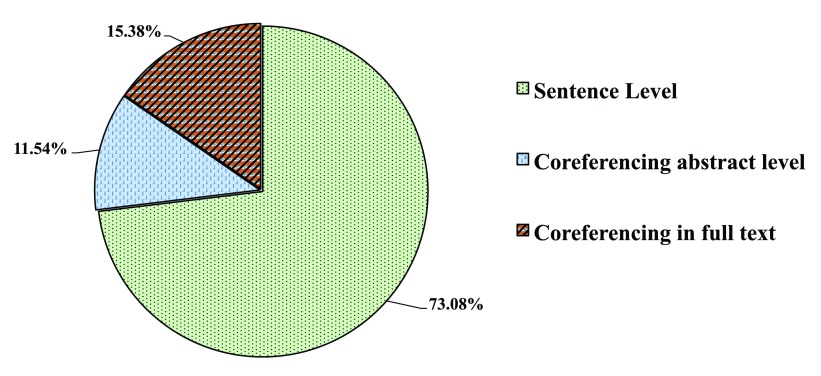
Coverage of relations occurring in Epilepsy Documents.
