# Supplementary material for: Detecting miRNA Mentions and Relations in Biomedical Literature
Source: F1000Res. 2015 Oct 1;3:205. Originally published 2014 Aug 28. [Version 3] doi: 10.12688/f1000research.4591.3 (PMC4602280; doi:10.12688/f1000research.4591.3)
Supplement: Supplementary file 1 [file f1000research-3-7643-s0000.tgz › 76d7fd67-9b67-4acd-861b-a09143edfe83_f1000research_corpus_suppl.zip!/Tables.pdf]

Table A. Count of entries available in the dictionaries.

	MeSH	MeSHAbbr	MedDRA	Genes/Proteins		Taxonomy		Relation Dictionary	
				Original	Processed	Original	Adapted	Dictionary	Spelling Variants
Entries	4,710	4,683	15,436	39,386	34,392	428,291	158	207	386
Synonyms	47,492	60,554	54,885	721,455	677,943	1,155,990	1,330	-	-

Table B. Performance evaluation of the disease dictionaries on training corpus (CM=Complete match and PM=Partial match).

	R		P		F_1	
	CM	PM	CM	PM	CM	PM
MeSH	0.62	0.74	0.70	0.70	0.66	0.72
MedDRA	0.50	0.59	0.73	0.86	0.60	0.70
MeSHAbbr	0.72	0.85	0.77	0.91	0.74	0.88

Table C. Results of tri-occurrence based approach for relation extraction using entities identified by ProMiner (NERTri).

	Training Corpus			Test Corpus		
Interacting Entity Classes	R	P	F_1	R	P	F_1
NonSpMiR-D	0.60	0.21	0.31	0.49	0.30	0.38
NonSpMiR-GP	0.53	0.35	0.42	0.87	0.36	0.51
SpMiR-D	0.62	0.31	0.41	0.52	0.38	0.44
SpMiR-GP	0.43	0.41	0.42	0.45	0.41	0.43

Table D. Comparison of different relation extraction approaches on test corpus.

	NonSpMiR-D			NonSpMiR-GP			SpMiR-D			SpMiR-GP		
RE approaches	R	P	F_1	R	P	F_1	R	P	F_1	R	P	F_1
Co-occurrence	1.00	0.20	0.34	1.00	0.28	0.44	1.00	0.41	0.58	1.00	0.53	0.69
Tri-occurrence	1.00	0.35	0.51	1.00	0.41	0.58	1.00	0.50	0.66	1.00	0.58	0.73
NERTri	0.49	0.30	0.38	0.87	0.36	0.51	0.52	0.38	0.44	0.45	0.41	0.43
Machine Learning Approaches												
LibLINEAR	0.51	0.56	0.53	0.64	0.57	0.60	0.73	0.62	0.67	0.87	0.68	0.76
SVM	0.49	0.55	0.52	0.63	0.56	0.59	0.73	0.61	0.66	0.86	0.68	0.76
Naive Bayes	0.60	0.52	0.56	0.75	0.57	0.65	0.65	0.62	0.63	0.90	0.64	0.75
