# Supplementary material for: Detecting miRNA Mentions and Relations in Biomedical Literature
Source: F1000Res. 2015 Oct 1;3:205. Originally published 2014 Aug 28. [Version 3] doi: 10.12688/f1000research.4591.3 (PMC4602280; doi:10.12688/f1000research.4591.3)
Supplement: Supplementary file 1 [file f1000research-3-7643-s0000.tgz › 76d7fd67-9b67-4acd-861b-a09143edfe83_f1000research_corpus_suppl.zip!/MiRNA_Annotation_Guidelines.pdf]

Annotation Guidelines for identification of specific miRNA mentions from text.

January 13, 2013

Annotation guidelines for specific miRNA mentions identification is generated with the help of observed facts during manual annotation and information obtained from some previous studies ([1], [2] and [3]).

Specific miRNA mentions annotation rules and examples (to be annotated are higlighted in bold):

1. MiRNAs mentions suffixed with numerical identifier (case insensitive) sould be annotated. For example, ***mir-21, MicroRNA-101, miR-101***, and ***MIR156***

2. Prefixed abbreviated species identifier must be annotated along with the miRNA terms, such as ***hsa-mir-100*** for *Homo sapiens* miRNAs.

3. Additional numerical suffix identifier are added for identical mature miRNA sequences originating from a different genomic loci in an organism which is to be annotated along with the miRNA mention, for instance, ***mir-6-1*** and ***mir-6-2***.

4. Prefixes used to describe more about miRNAs mentions, such as, "mature-" or "pri-" or "primary-" or "pre-", are ignored and only the miRNA mention text is to be annotated, for example, *pri-**miR-132***, and *mature-**miR-132***.

5. Two microRNA species arising from different miRNA hairpin precursors are mentioned with additional suffixes 5p/s and 3p/as (represent 5-arm and 3-arm), annotate ***hsa-miR-1224-5p***, ***hsa-miR-1224-3p***, ***miR-142-s***, and ***miR-142-as***.

6. Annotate less informative/predominant form of miRNA mention suffixed with an asterix, such as, ***hsa-miR-100****.

7. Similar miRNA sequences (that arise from same loci) with just one or two base changes are represented by an additional sequential alphabet suffix, for instance ***miR-181a*** and ***miR-181b***. At times both sequences are mentioned together, for example, ***miR-126a/b***. In such cases, annotate the complete string, *e.g.* ***miR-126 and b*** or ***miR19a and a9b***.

8. Annotate miRNA cluster mentions, *e.g.* ***miR-23a∼27a∼24-2***.

9. Numerical identifiers as part mentions need to be annotated as a single instance, for example, ***MicroRNAs 1, 133, and 206***.

10. Similar mature microRNA sequences arising from different genes are assigned different identifiers which needs to be annotated as a single instance, for example, ***mir156/157***.

11. Synonyms of cluster names needs to be annotated, for example, ***miR-17-92*** cluster (***oncomir-1***).

12. Two separate instances of miRNA mentions separated by delimiters (",", "/", and "and") needs to be annotated separately, for example, ***mir-34b***/***mir-34c***, ***mir-34b*** and ***mir-34c***.

13. Annotate complete text even if miRNA term is mentioned as a substring, *e.g.* ***(miR)-223***.

14. Some exceptions in miRNA terms such as, ***miR-N367***, ***let-7***, and ***lin-4*** , should additionally be annotated.

Specific microRNA mentions annotation rules and examples (not to be annotated):

1. Non informative or general terms like *microRNAs*, and *miRNA*, should not be annotated.

2. If miRNA is prefixed by "*anti*", "*antisense*" or "*antisense oligonucleotide*", for example, *antisense oligonucleotide miRNA-146a* (*anti-miRNA-146a*; AM-146a), should not be annotated.

References

[1] Naeem, Haroon and Küffner, Robert and Csaba, Gergely and Zimmer, Ralf (2010).miRSel: automated extraction of associations between microRNAs and genes from the biomedical literature. *BMC Bioinformatics*, **11**,135.

[2] Griffiths-Jones, Sam (2004). The microRNA Registry. *Nucleic Acid Research (Database Issue)*, **32**, D109–11.

[3] Ambros, V. (2003). A uniform system for microRNA annotation. *RNA*, **9(3)**:277279.
